# Crescentic Glomerulonephritis, A Rare Presentation of Alport Syndrome

**DOI:** 10.30699/IJP.2022.554160.2899

**Published:** 2022-09-02

**Authors:** Tahereh Malakoutian, Fatemeh Nili, Sholeh Ghasemi Darbrood, Samaneh Salarvand, Mitra Mehrazma

**Affiliations:** 1 *Department of Nephrology, Shahid Hasheminejad Kidney Center, Iran University of Medical Sciences, Tehran, Iran*; 2 *Department of Pathology, Imam Khomeini Hospital Complex, Tehran University of Medical Sciences, Tehran, Iran*; 3 *Department of Pathology, Shahid Hasheminejad Kidney Center, Iran University of Medical Sciences, Tehran, Iran*

**Keywords:** Alport, Crescentic glomerulonephritis, Hereditary nephritis

## Abstract

Crescentic glomerulonephritis (GN) is a feature of severe glomerular injury. Anti-GBM disease, immune-complex mediated glomerulonephritis, and ANCA-associated vasculitis are the main causes of crescentic GN. Alport syndrome is a progressive form of hereditary nephritis presenting with hematuria and progression to proteinuria and renal failure. Herein we present a 16-year-old male with rapidly progressive glomerulonephritis syndrome, sensory-neural hearing loss, and a family history of hematuria and proteinuria in his mother and aunt. Light microscopic examination shows cellular crescent in glomeruli. In an electron microscopy study, GBM changes compatible with Alport syndrome were identified. Alport syndrome rarely can be presented as crescentic GN. Electron microscopy is necessary for the diagnosis of this type of pauci-immune crescentic glomerulonephritis.

## Introduction

Crescentic glomerulonephritis presenting as rapidly progressive glomerulonephritis (RPGN) syndrome is typically associated with severe forms of inflammatory glomerular injury (1, 2). They are subdivided into 5 subcategories according to immunofluorescence and serologic findings. Type 1 is associated with the anti-GBM disease. Type 2, is also associated with immune complex-mediated glomerulonephrites (GN) such as IgA nephropathy, lupus nephritis, MPGN, or post-infectious GN. In type 3, no immune reaction in IF study is seen and associated with positive ANCA. Type 4 is a combination of type 1 and type 3. Type 5 are the cases of pauci-immune crescentic GN with negative ANCA (1).

Alport syndrome is a type of hereditary nephritis that usually involves male patients because of the X-liked inheritance pattern (3). The most common clinical presentation is hematuria with gradual progression to proteinuria and renal failure (4). RPGN and crescentic GN is a very rare manifestation of Alport syndrome, with few reported cases in the literature.

##  Case Report

A 16-year-old male without any systemic disease went to the doctor's office to complain of occasional headaches, which started 6 months ago.

The headache pattern was irregular in day and night without nausea, vomiting, or focal neurologic deficit. He also said mild pitting edema in lower extremities, limited to one-third of the calves' distal part. He reported mild hearing loss in both ears a year before his referral, with a sensory-neural pattern in pure tone audiometry. At the first clinical visit, he had high blood pressure (150/90). Laboratory results are as follow: WBC: 13400/L, Hemoglobin: 10.5mg/dl, Platelets: 243000/L

Blood Urea Nitrogen: 185, Creatinine: 2.9 mg/dL, Urine Analysis: protein 3+, blood: 3+, RBC: 16-18 /LPF, granular cast: 2-3/LPF

24 h urine volume: 1400cc, 24 h Creatinine: 590 mg, 24 h protein: 998 mg, weight: 58 kg

In secondary work-up, he had a normal level of Complements 3. Other serologic tests were normal. He informed us about his mother and aunt's history of proteinuria and hematuria. They didn't have been evaluated. The patient was treated with 25 mg daily prednisolone and losartan 25 twice daily and referred to our hospital for further evaluation. On physical examination, stable vital signs, high blood pressure, and mild pitting edema (up to 1+) were detected in the lower extremities. He didn't complain of any oliguria. Ophthalmologic examination revealed normal fundus and retina. Other systems were also normal. The patient underwent 2 sessions of hemodialysis, and then a kidney biopsy was taken.

**Fig. 1:**
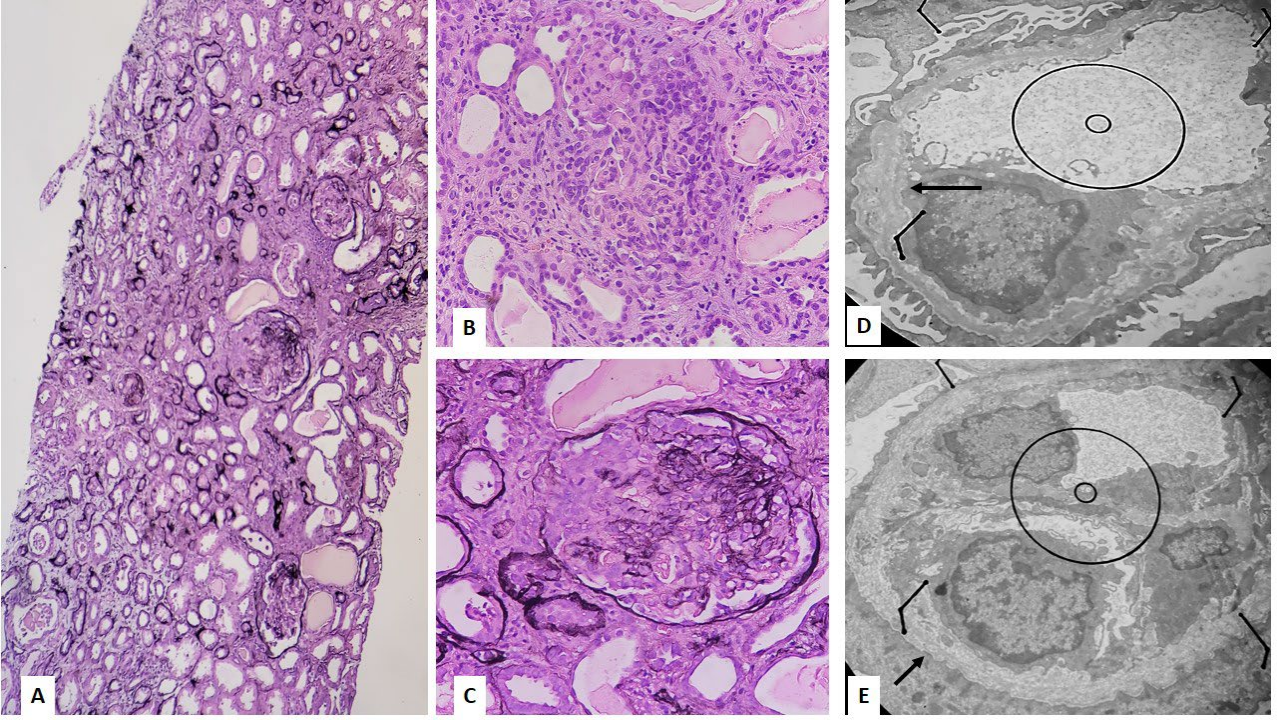
Microscopic examination showing cellular crescent, rupture of Bowman capsule and GBM, interstitial inflammation, tubular atrophy, and interstitial fibrosis (A, C: Jone's methenamine silver stain, B: H&E stain, 100X, 400X). The ultrastructural evaluation showing irregular GBM with lamellation of lamina densa, basket weave appearance, and scalloping at the epithelial side (D, E, 5800X)

Light microscopic examination of renal biopsy revealed corticomedullary kidney tissue containing 13 glomeruli with global sclerosis in two and cellular crescent in nine other glomeruli. The remaining preserved glomeruli were unremarkable. In the interstitium, mild infiltration of chronic inflammatory cells and about 35% fibrosis were seen. Proportional tubular atrophy and RBC casts in the tubules were also identified. Vessels show normal structure (Fig.1.A-C). Immunofluorescence study for IgG, IgA, IgM, C3c, C4c, C1q, and Albumin didn't show glomerular or tubular staining. Fibrinogen highlighted cellular crescents. On Electron microscopy, global effacement of visceral foot processes, diffuse GBM thickening with lamellation of lamina densa, scalloping at the epithelial site, and basket waeve appearance with segmental foci of thinning, tearing, or rupture were seen. The proliferation of parietal epithelial cells, necrosis, and mononuclear inflammatory cell infiltration making cellular crescent, were also evident (Fig.1.D, E).

## Discussion

Alport syndrome is a progressive form of hereditary nephritis that develops due to mutations in COL4A3, COL4A4, and COL4A5 genes encoding α3, α4, and α5 chains of collagen type 4 (3). Defects in the assembly of α chains of collagen lead to a break in the basement membrane of glomeruli, cochlea, and the base of the ocular lens. Mutation of COL4A5 with an X-linked pattern of inheritance is the most frequent form of the disease (80%). The other cases show mutations of COL4A3 or COL4A4 and are inherited as autosomal recessive (15%) or autosomal dominant (5%) diseases (5, 6).

Based on the Japanese society of pediatric nephrology working group on Alport syndrome (revised in February 2015), the main criteria for diagnosis is the history of persistent hematuria when the patients show one or more secondary features (Type 4 collagen abnormal expression, mutations in type 4 collagen genes or specific GBM changes on Electron microscopy) or two or more accessory features (family history of kidney disease, bilateral sensorineural deafness, ocular abnormalities or diffuse leiomyomatosis) they can be diagnosed as Alport syndrome (3). 

There is no specific pathology finding on light microscopy. Mesangial hypercellularity, segmental sclerosis, interstitial fibrosis, and aggregates of foam cells have been reported. The specific electron microscopy findings include irregularity of GBM thickness, lamellation and splitting of lamina densa (7).

Crescentic glomerulonephritis is characterized by proliferating parietal epithelial cells with necrosis and infiltration of macrophages beneath the Bowman capsule in more than 50% of glomeruli. It is a feature of severe glomerular injury. The crescent formation is a nonspecific response to severe injury of the glomerular capillary wall, its rupture, or physical gaps. This type of injury permits the entry of coagulation factors into the Bowman's space, fibrin formation, activation, and proliferation of epithelial cells. Anti-GBM disease, immune-complex mediated glomerulonephritis, and ANCA-associated vasculitis are the main causes of crescentic GN. About 5-10% of the cases are pauci-immune ANCA-negative vasculitis (1).

Alport syndrome with severe damage to the GBM, and increased pressure of the capillaries, may rarely present with glomerular crescents. Chugh* et al.* reported 1 out of 63 cases of Alport syndrome with crescent (8). Afonso *et al.* reported a 20% frequency of crescent in Alport syndrome patients (9). Other case reports from Harris* et al.* and Halder* et al.* also described this rare manifestation of Alport syndrome (10, 11).

The rare cases reported in the literature and possible pathogenetic factor of GBM damage and crescent formation in Alport syndrome highlight the significance of electron microscopic examination in pauci-immune types of crescentic glomerulonephritis.

## Conclusion

Alport syndrome rarely can be presented as crescentic GN. Electron microscopy is necessary to diagnose this type of pauci-immune crescentic glomerulonephritis.

## Ethics approval & Consent to Participate

Informed constant was obtained from the patient.

## Authors' contributions

T. M: case presentation and management, F. N: pathology diagnosis and preparation of the manuscript, SH. Gh: case presentation and preparation of the manuscript, S. S, M. M: pathology diagnosis and preparation of the manuscrpit

## Conflict of Interest

The authors declared no conflict of interest.

## Funding

The author(s) received no financial support for the research, authorship, and/or publication of this article.

## References

[1] Parmar MS, Bashir K (2020). Crescentric glomerulonephritis.

[2] Hénique C, Papista C, Guyonnet L, Lenoir O, Tharaux P-L (2014). Update on crescentic glomerulonephritis. Seminars in immunopathology.

[3] Nozu K, Nakanishi K, Abe Y, Udagawa T, Okada S, Okamoto T (2019). A review of clinical characteristics and genetic backgrounds in Alport syndrome. Clin Exp Nephrol.

[4] Wester DC, Atkin CL, Gregory MC (1995). Alport syndrome: clinical update. J Am Acad Audiol.

[5] Feingold J, Bois E, Chompret A, Broyer M, Gubler MC, Grunfeld JP (1985). Genetic heterogeneity of Alport syndrome. Kidney Int.

[6] Tryggvason K, Zhou J, Hostikka SL, Shows TB (1993). Molecular genetics of Alport syndrome. Kidney Int.

[7] Haas M (2009). Alport syndrome and thin glomerular basement membrane nephropathy: a practical approach to diagnosis. Arch Pathol Lab Med.

[8] Chugh K, Sakhuja V, Agarwal A, Jha V, Joshi K, Datta B (1993). Hereditary nephritis (Alport's syndrome)-clinical profile and inheritance in 28 kindreds. Nephrol Dial Transplant.

[9] Afonso A, Valente I, Macedo L, Sampaio S, Faria S, Costa T (2010). Alport syndrome-a rare histological presentation. Port J Nephrol Hypert.

[10] Harris J, Rakowski T, Argy Jr W, Schreiner G (1978). Alport's syndrome representing as crescentic glomerulonephritis: a report of two siblings. Clin Nephrol.

[11] Haldar I, Jeloka T (2020). Alport's Syndrome: A Rare Clinical Presentation with Crescents. Indian J Nephrol.

